# Patient-Led Lifestyle Questionnaire to Help Improve Lifestyle Interventions Offered to Patients

**DOI:** 10.1192/bjo.2024.601

**Published:** 2024-08-01

**Authors:** Kuljit Mandair, Louisa James

**Affiliations:** 1 Black Country Healthcare Foundation NHS Trust, Black Country, United Kingdom; 2 Hereford and Worcestershire Healthcare NHS Foundation Trust, Worcestershire, United Kingdom

## Abstract

**Aims:**

Maintaining a healthy lifestyle plays a vital role in the prevention and management of many mental conditions. There is also evidence that these patients have a lesser standard of health promotion and physical care and despite national awareness and guidelines early mortality rates have not improved.

The aim of this audit cycle was to firstly establish whether lifestyle interventions are being offered to patients (in a Home Treatment Team) and secondly how could this be further improved. A patient-led lifestyle intervention was introduced whereby the aim was to help patients feel empowered by being able to select an area of lifestyle they would like to improve. A coaching style framework was used and the patient was assisted in setting a lifestyle related goal to help with their mental health recovery.

**Methods:**

An audit was carried out on 20 Physical Health Forms in January 2023 looking at the documentation of lifestyle interventions offered in the following lifestyle domains: smoking, alcohol, substance misuse, diet, exercise and the measuring of waist circumference and weight. This is a form that is usually completed by Psychiatric nurses based in the Worcester South Home Treatment Team during initial patient assessments.

The audit showed low levels of interventions offered to patients for lifestyle domains and therefore staff education on the importance of lifestyle and the importance of measuring waist circumference was delivered within a team meeting setting. A patient led lifestyle questionnaire was also initiated. After implementing this for 3 months, a re-audit was completed of 20 physical health forms in May 2023.

**Results:**

The re-audit results showed an increase in lifestyle interventions offered to patients in all lifestyle domains. There was a 30% increase in patients being offered interventions in exercise, a 40% increase in patients being offered interventions in diet, 20% increase in patients having waist circumference measured, 5% increase in patients being offered substance misuse interventions, 10% increase in patients being offered interventions for alcohol misuse and a 30% increase in patients being offered interventions for smoking.

**Conclusion:**

There is growing evidence that by addressing lifestyle factors we can improve overall patient care outcomes by raising awareness and including lifestyle modification to be a part of the treatment plan. Using a coaching framework can be an effective part of the management plan by helping patients to feel empowered and future focused to improve their lifestyle and therefore their own health.

